# Decreased peak atrial longitudinal strain and left atrial contraction strain rate values may predict atrial fibrillation in patients in sinus rhythm at baseline: a systematic review and meta-analysis

**DOI:** 10.1093/ehjopen/oeaf024

**Published:** 2025-03-20

**Authors:** Henriette Mészáros, Rita Nagy, Péter Fehérvári, Péter Hegyi, Enikő Pomozi, Zsombor Simon, Andrea Ágnes Molnár, Sándor Nardai, Béla Péter Merkely, Pál Ábrahám

**Affiliations:** Heart and Vascular Centre of Semmelweis University, Városmajor Street 68, 1122 Budapest, Hungary; Centre for Translational Medicine, Semmelweis University, Baross Street 22, 1085 Budapest, Hungary; Centre for Translational Medicine, Semmelweis University, Baross Street 22, 1085 Budapest, Hungary; Heim Pál National Pediatric Institute, Üllői Way 86, 1089 Budapest, Hungary; Institute for Translational Medicine, University of Pécs, Szigeti Way 12, 7624 Pécs, Hungary; Centre for Translational Medicine, Semmelweis University, Baross Street 22, 1085 Budapest, Hungary; Centre for Translational Medicine, Semmelweis University, Baross Street 22, 1085 Budapest, Hungary; Institute for Translational Medicine, University of Pécs, Szigeti Way 12, 7624 Pécs, Hungary; Division of Pancreatic Diseases, Heart and Vascular Center, Semmelweis University, Tömő Street 25-29, 1083 Budapest, Hungary; Heart and Vascular Centre of Semmelweis University, Városmajor Street 68, 1122 Budapest, Hungary; Centre for Translational Medicine, Semmelweis University, Baross Street 22, 1085 Budapest, Hungary; Centre for Translational Medicine, Semmelweis University, Baross Street 22, 1085 Budapest, Hungary; Heart and Vascular Centre of Semmelweis University, Városmajor Street 68, 1122 Budapest, Hungary; Heart and Vascular Centre of Semmelweis University, Városmajor Street 68, 1122 Budapest, Hungary; Centre for Translational Medicine, Semmelweis University, Baross Street 22, 1085 Budapest, Hungary; Centre for Neurosurgery and Neurointervention, Semmelweis University, Laky Adolf Street 44, 1145 Budapest, Hungary; Heart and Vascular Centre of Semmelweis University, Városmajor Street 68, 1122 Budapest, Hungary; Centre for Translational Medicine, Semmelweis University, Baross Street 22, 1085 Budapest, Hungary; Heart and Vascular Centre of Semmelweis University, Városmajor Street 68, 1122 Budapest, Hungary; Centre for Translational Medicine, Semmelweis University, Baross Street 22, 1085 Budapest, Hungary

**Keywords:** Speckle-tracking echocardiography, Left atrial strain, PALS, Atrial cardiomyopathy, Atrial fibrillation

## Abstract

Despite the broadening spectrum of heart rhythm-monitoring techniques, the most appropriate modality and duration to detect atrial fibrillation (AF) are not consensual. Two-dimensional speckle tracking for left atrial strain analysis seems feasible to detect atrial cardiomyopathy, which represents a substrate for AF. We aimed to perform a systematic review and meta-analysis on peak atrial longitudinal strain (PALS) and contraction strain rate (pLAct) differences as primary outcomes measured in sinus rhythm (SR) between patients with and without future AF during follow-up (FU). Random-effect model was used. For continuous outcomes, we calculated weighted mean differences (WMD) to compare the two groups We identified eight eligible articles consisting of 1230 patients. Six articles were eligible for quantitative PALS analysis, while three were eligible regarding pLAct. The baseline parameters of the future AF and SR patients were comparable in most of the papers. The PALS value in patients developing future AF was significantly lower compared to patients with no AF (WMD: −5.55% 95% CI −7.06 to −4.03%). Pooled WMD of pLAct between the groups was −0.44 1/s, 95% CI: −0.56 to −0.33 1/s. Baseline PALS and pLAct values of future AF patients were significantly lower than those who remained in SR during FU. PALS and pLAct seem to be sensitive parameters which can be implemented in a predictive model for AF enabling us to find patients who need prolonged heart rhythm-monitoringand it can be also a guidance for oral anticoagulation indication setting.

## Introduction

Ischaemic stroke (IS) is one of the leading causes of disabilities worldwide.^1^ Identifying the stroke mechanism is important to properly reduce the risk of recurrent events. Despite thorough diagnostic evaluation, the source remains unknown in cryptogenic cases, and 20–30% of these are thought to be associated with atrial fibrillation (AF).^2^ According to recent estimations, this arrhythmia affects 2–4% of the global population,^3^ and we also expect a large rise in the diagnosed numbers, because of the broadening spectrum of rhythm-monitoring modalities.^4^ Owing to the nature of this arrhythmia (often asymptomatic and paroxysmal), it poses a big diagnostic challenge to document it with electrocardiogram (ECG).^5^ Moreover, asymptomatic AF is a higher risk factor for suffering IS compared with the symptomatic type.^6^ According to guideline recommendations, for the diagnosis of AF, we must have the arrhythmia documented with a 12-lead ECG or an episode lasting 30 s or longer on a single-lead rhythm-monitoring device.^7^ These facts also emphasize the importance of screening for AF. Prolonged ECG-monitoring strategies are promising diagnostic tools; however, neither the right method, nor the appropriate duration of these time-consuming procedures are precisely defined, and there is no wide consensus on it.^8^ Several wearable heart rhythm-monitoring strategies via smartphones are rapidly developing nowadays and provide easily available monitoring options.^9^ However, we have to be aware of the fact that some of them are not clinically validated or not registered as a medical diagnostic device.^10^ The two main studies with smartphone apps are the Apple Heart Study and Huawei Heart study, which concluded that clinically validated smartphone apps using continuously functioning photoplethysmography, which triggers an alert for performing a single-lead ECG are feasible for home AF monitoring.^11,12^. However the arsenal of smart-monitoring techniques is broad and are reliable in most of the cases, but they can barely compete with implantable cardiac-monitoring devices (ICM-D). The CRYSTAL-AF study stated that the AF detection rate of ICM-D is much higher, than of the conventional 24-h Holter ECG monitoring.^1^ The NOAH-AF NET study^13^ published last year found, that the initiation of oral anticoagulation (OAC) driven by short atrial high rate episodes (AHRE) in ICM-D did not reduce the composite endpoint of major cardiovascular events (MACEs), but significantly increased the risk of major and fatal bleeding. Although ARTESIA Trial from this year found^14^ that Apixaban reduced the risk of MACE in this AHRE patient population when compared with Aspirin, but similarly increased the risk of major bleeding.

As seen, despite the broadening spectrum of internal- and external-monitoring devices, there is still no consensus on which modality and duration should be used in clinical practice to detect AF episodes with stroke relevance. A paradigm shift can be observed, which suggests to consider the use of AF burden rather than the binary definition of AF when talking about AF-related outcomes.^15^ The EAST-AFNET^16^ was the first study to state, that early rhythm control therapy is associated with a lower risk of the composite MACE endpoint which included ischaemic stroke. More accurate selection of patients who would benefit from early rhythm control could be enhanced by the use of AF burden and the detection of undelying ACM. Our idea could help the first identification and the later treatment of AF, if we could find and implement a non-invasive, yet sensitive method to evaluate the risk of clinically significant AF episodes with a single measurement of atrial function.^17^

In 90% of AF-related strokes the source of embolism is the left atrium (LA). LA deformation imaging and strain analysis by two-dimensional speckle-tracking echocardiography for assessing the morphological and functional dimensions of atrial cardiomyopathy (ACM) is a promising non-invasive technique with a potential to detect the underlying structural and functional changes creating the substrate for AF. ACM is basically a consequence of LA remodelling, which is an adaptation of the cardiac myocytes to systematic- or cardiac-derived causes (e.g. hypertension (HT), diabetes mellitus (DM), congestive heart failure).^18^ According to newest literature data, ACM is present not only on the cellular electrophysiological level, but also in structural and contractile changes on the cardiomyocyte level.^19^ There is evidence based data from patients with cardiac implantable electronic devices, that the risk of IS is increased mainly by the comorbidities and age expressed with the CHA_2_DS_2_-Vasc score, and not by the duration of AF only. The latter counts for more strokes only in the higher score ranges of 2–5. These comorbidities are responsible for developing ACM.^20^

LA strain measurement provides an advanced assessment of the LA mechanics, deformation and underlying ACM. This modality eliminates the limitations (e.g. fore-shortening, lack of evidence for LA functional assessment), which come with using the conventional two-dimensional echocardiographic LA measurements.^21^ Recognition of LA remodelling is dilation of the LA, which leads to increased left atrial stiffness.^22^ It remains unclear whether this diagnostic method should be used for screening in patients who are at risk for developing AF. To fill this knowledge gap, we aimed to provide a detailed analysis of the currently available eligible studies assessing the diagnostic yield of different LA deformation imaging parameters regarding future AF development.

## Methods

The review protocol for this systematic review and meta-analysis was prospectively registered with PROSPERO (CRD42021286775). Findings are reported in accordance with the Preferred Reporting Items for Systematic Reviews and Meta-analyses (PRISMA) reporting guideline.^23^

### Study selection

A systematic search was carried out in the following databases: MEDLINE (via PubMed), Embase, Cochrane Central Register of Controlled Trials (CENTRAL), Web of Science and Scopus. The search was conducted on 8 November 2021. We did not use any restrictions. Search key was the following: (‘atrial fibrillation’ OR ‘AF’) AND (‘LA strain’ OR ‘speckle-tracking echocardiography’ OR ‘speckle tracking echocardiography’ OR ‘atrial strain’ OR ‘atrial dysfunction’ OR ‘atrial cardiopathy’ OR ‘atriopathy’). The selection process was conducted by two independent reviewers according to a predefined set of rules with the help of a reference management software (Endnote X9 software; Clarivate Analytics; 2019). Removal of duplicates was first performed automatically and manually thereafter. Records were screened based on title, abstract, and full text. After each step of the selection process, the rate of agreement was determined and documented by calculating the Cohen κ coefficient. Values may indicate slight (0–0.20), fair (0.21–0.40), moderate (0.41–0.60), substantial (0.61–0.80), and almost perfect agreement (0.81–1.00). Disagreements were resolved by consensus.

For data extraction, we created a standardized data collection sheet. The following data was extracted from each eligible article: title of the study, publication year, DOI identification, age distribution, number of patients in each comparison group, number of patients in each event, mean or median values in the group of our outcomes of interest.

### Eligibility criteria

Cohort studies, randomized controlled trials (RCTs), conference abstracts were eligible. Animal studies, case reports, systematic reviews or studies with no original data were excluded. The research question was formulated using the Population, Exposure, Comparator, and Outcomes framework.^24^

We included studies dealing with cardiology patients above the age of 18 years, who underwent LA strain analysis using two-dimensional speckle-tracking echocardiography in SR and were monitored for AF during the follow-up. Exclusion criteria were previous intervention (e.g. catheter ablation) on the atria or AF detection at baseline.

Primary outcomes included LA strain (peak atrial longitudinal strain PALS), LA strain rate (contraction strain rate pLAct), because these are the two most common used strain parameters.

### Data extraction

Two cardiology fellows (H.M. and E.P.) independently extracted data into a standardized data collection sheet (Excel 2019; Microsoft Corp). Data collection included sex distribution, age distribution, patient numbers, and mean or median values of outcomes of interest.

### Risk of bias assessment

Based on the recommendations of the Cochrane Prognosis Methods Group, the Quality in Prognostic Studies (QUIPS) tool was applied by two of us (H.M. and E.P.) to assess the risk of bias in the included studies for each outcome separately.^32^ Any disagreement was resolved based on consensus. Further details regarding Risk of bias assessment (RoB) can be found in results part.

### Statistical analysis

Depending on the data heterogenity random-effect model was used. For continuous outcomes, we calculated mean differences to investigate the differences between the two groups. We assessed statistical heterogeneity using Cochrane’s *Q* and *I*² statistics. Statistical tests were performed at the 0.05 significance level. Heterogeneity was considered significant if *P*-value < 0.1. Analyses were performed with the use of R (Ross Ihaka and Robert Gentleman, New Zealand, 1993), a language and environment for statistical computing.

### Primary outcomes

#### GLAS – global longitudinal atrial strain (%)/PALS – peak atrial longitudinal strain (%)

All eligible studies reported GLAS and PALS (peak atrial longitudinal strain) as an indicator of left atrial mechanical function. Although we are fully aware of the slightly different definitions of GLAS and PALS, because of the same measurement points on the LA strain curve exhibiting LA reservoir function, we regarded PALS and GLAS measurements as the same variables. According to the task force of Badano et al.,^25^ LA global longitudinal strain should be calculated as the longitudinal strain obtained from a non-foreshortened apical four-chamber view. Biplane LA longitudinal strain should be available as an option. Both PALS and GLAS represent the reservoir function of the LA. For a simplified nomenclature, we will use PALS abbreviation both for GLAS and PALS in the further parts of the article.

#### pLAct rate – atrial contraction strain rate (1/s)

The eligible studies^26–28^ for the assessment of pLAct reported this variable as an indicator of left atrial booster pump function. The same study mentioned above was included here also only in the qualitative synthesis, because of the same reason detailed before.

### Secondary outcomes

#### pLAct − peak atrial contraction strain (%)

Peak atrial contraction strain (PACS) is measured during contraction phase, in patients with sinus rhythm, it displays the difference of the strain value at ventricular end-diastole minus at the onset of atrial contraction.^25^

#### Atrial reservoir strain rate (1/s) and atrial conduit strain rate (1/s)

Atrial reservoir strain rate (Srs) is myocardial deformation speed during reservoir phase, measured as the difference of the strain value at mitral valve opening minus ventricular end-diastole. Atrial conduit strain rate (Sre) is myocardial deformation speed during conduit phase, measured as the difference of the strain value at the onset of atrial contraction minus mitral valve opening.^25^

#### LA stiffness index (ratio of *E/e*′ to sr)

The LA stiffness index is the ratio of early mitral inflow velocity-to-early annular tissue velocity (*E/e*′, by Doppler ultrasound examination) and LA reservoir strain.^31^ Both studies reported increased baseline stiffness of the LA wall affecting patients with AF.

## Results

### Study characteristics

**Figure 1:**
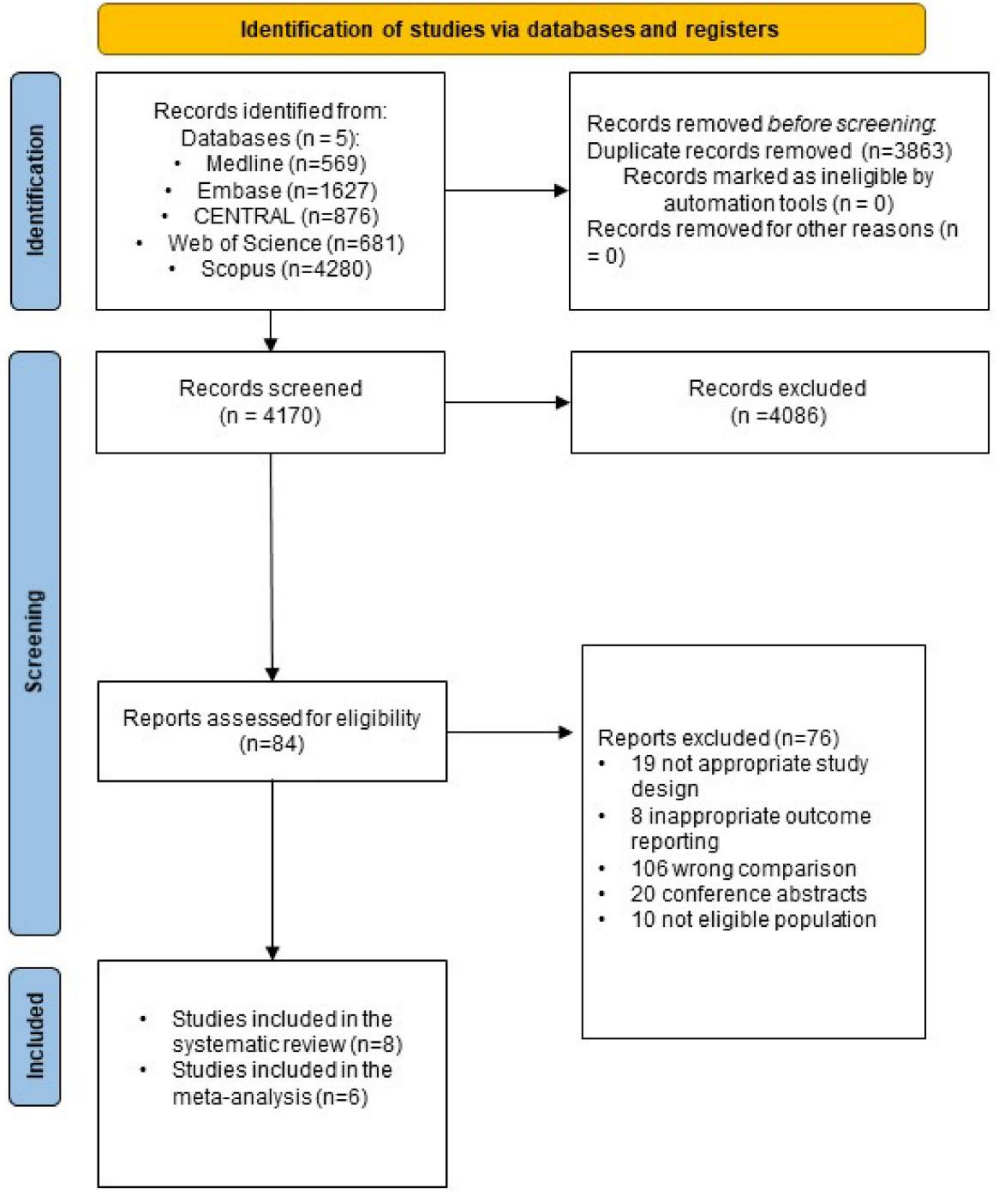
PRISMA flowchart of the selection process.

Altogether, 1230 patients were included in the systematic review and meta-analysis from a total of eight studies between 2011 and 2021. Five^26,27,29–31^ of eight studies were prospective studies and three^28,32,33^ were based on retrospective observations. The vast majority of the studies^26–28,30–33^ were single-centre observations, with the exception of one^32^ multicentre study. Seven studies^26–32^ comprising data of 1136 patients were eligible for performing comprehensive meta-analysis regarding left atrial strain and strain rate outcomes. All data is detailed in *Figure 1*. All patients were in sinus rhythm at the time of left atrial strain measurements, as this was a fundamental issue in our study design.

### Patient characteristics

Although the overall study population was heterogeneous ranging from poststroke patients to those awaiting cardiac surgery, all included patients had no previous surgical- or catheter-based intervention affecting their LA at baseline. The most important published patient characteristics of the studies such as gender distribution, age, prevalence of HT, DM and the value of left ventricular ejection fraction were also summarized. All assessed parameters were comparable among those who developed AF during follow-up and those remaining free from AF in the papers, except for^33^ and,^26^ where patients with AF had significantly more HT. In the article of Hirose et al^26^ and Basaran et al,^30^ age was significantly higher in patients who developed AF during the follow-up period. Pooled patient-level data analysis of the eight studies could not be performed because of no access to individual patient databases. Characteristics of all included studies are shown in *Table 1*.

**Table 1:** Baseline characteristics of studies included in systematic review and meta-analysis

Study author	Study design	AF detection	AF group demography						Control group demography						Outcome	AF-monitoring time
			Population (*n*)	Age Mean ± SD	Number of males (%)	Number of patients with HT (%)	Number of patients with DM (%)	LV EF Mean ± SD	Population (*n*)	Age Mean ± SD	Number of males (%)	Number of patients with HT (%)	Number of patients with DM (%)	LV EF Mean ± SD (%)		
**Hirose et al., 2011, Japan**	Prospective	Monthly in-office ECG regardless of symptoms	32	72.9 ± 9^a^	18 (56.3)	25 (78.1)^d^	3 (9.4)	64 ± 7	548	64.1 ± 17	285 (52)	297 (54.2)	45 (8.2)	65 ± 8	PALS, pLAct rate	monthly ECG examinations, AF detection: (median: 28 months, 25 percentile: 26 months, 75%ile: 30 months)
**Hong et al., 2013, China**	Retrospective	According to guideline recommendation	45	51.8 ± 6.8	30 (67)	N/A	N/A	68.07 ± 6.26	30	48.97 ± 7.65	18 (60)	N/A	N/A	65.4 ± 5.51	PALS, pLAct rate	ECG by enrolment and follow-up
**Pagola et al.,2014, Spain**	Case–control, retrospective	72-h monitoring	34	N/A	18 (52.9)	16 (47.1)	6 (17.6)	N/A	13	N/A	8 (61.5)	7 (53.8)	4 (30.8)	N/A	PALS	3-year cardiac monitoring with Holter Reveal Tx
**Pagola et al., 2020, Spain**	Prospective	Holter (CryptoAF registry pattern)	pAF <5 h: 20^c^	73.6 ± 7.6	18 (52.9)	15 (75)	4 (22.2)	N/A	199	73.6 ± 9.3	101 (50.75)	144 (77)	43 (24)	N/A	PALS	Holter device (NuuboR) for 28 days
			pAF >5 h: 34^c^	79.9 ± 7.1	79.9 (7.1)	29 (85.3)	11 (35.5)	N/A								
**Imanishi et al., 2013, Japan**	Prospective	ECG monitoring	15	78 ± 10	3 (20)	13 (87)	4 (27)	61 ± 13	12	75 ± 6	5 (41.67)	10 (83)	3 (25)	66 ± 8	PALS, pLAct rate	14.5 ± 5.7 days following surgery
**Basaran et al., 2016, Turkey**	Prospective	4-day monitoring, then daily ECG	23	64.3 ± 7.4^b^	16 (69)	9.9 (43)	6 (26)	N/A	67	58.7 ± 10.1	55 (82)	25 (37)	17 (25)	N/A	PALS	4 days in the intensive care unit and step-down unit. Postoperatively, 12 lead ECG were obtained daily. Postoperative AF was defined as any episode of AF lasting at least 15 min
**Lin et al.,2021, China** ^e^	Prospective	According to 2014 AHA guideline recommendation	27	54.8 ± 10.3	40 (59.8)	9 (33.3)	6 (22.2)	54.8 ± 6.2	67	48.8 ± 7.5	40 (59.3)	20 (29.8)	8 (11.9)	55.7 ± 6.8	PALS, pLAct rate	ECG by enrolment and follow-up

AF, atrial fibrillation; AHA, American Heart Association; DM, diabetes mellitus, HT, hypertension; LV EF, left ventricular ejection fraction; PALS, peak atrial longitudinal strain; pLAct rate, contraction strain rate;

^a^p < 0.05 vs. control group (*P* = 0.004).

^b^ *P* < 0.05 vs. control group (*P* < 0.001).

^c^ *P* < 0.05 vs. control group (*P* = 0.01; Kruskal–Wallis test).

^d^ *P* < 0.05 vs. control group (*P* = 0.008).

^e^Only included in the systematic review.

### Risk of bias assessment

We used the QUIPS tool to assess the risk of bias regarding the included studies. Regarding PALS outcome, four out of six^26,28,30,32^ carry moderate risk for bias, the other two,^29,33^ have low risk of bias.

Mostly moderate risk of bias were found also regarding pLAct rate, two studies^26,28^ seemed to be moderate risk of bias, while one^27^ carries low risk of bias.

Further details regarding RoB can be found in *Tables 2 and 3*.

**Table 2:** Risk of bias assessment (RoB) QUIPS outcome 1: PALS (%)

Study	Study participation	Study attrition	Prognostic factor measurement	Outcome measurement	Study confounding	Statistical analysis reporting	Overall risk of bias
Hirose et al. 2011	low risk of bias	low risk of bias	low risk of bias	low risk of bias	high risk of bias	low risk of bias	moderate risk of bias
Hong et al. 2013	moderate risk of bias	moderate risk of bias	moderate risk of bias	moderate risk of bias	moderate risk of bias	low risk of bias	moderate risk of bias
Pagola et al. 2014	moderate risk of bias	moderate risk of bias	moderate risk of bias	low risk of bias	moderate risk of bias	low risk of bias	moderate risk of bias
Pagola et al. 2021	low risk of bias	low risk of bias	low risk of bias	low risk of bias	moderate risk of bias	low risk of bias	low risk of bias
Basaran et al. 2015	moderate risk of bias	moderate risk of bias	moderate risk of bias	moderate risk of bias	moderate risk of bias	low risk of bias	moderate risk of bias
Rahman et al. 2014	moderate risk of bias	low risk of bias	moderate risk of bias	low risk of bias	low risk of bias	low risk of bias	low risk of bias
Total low	2	3	2	4	1	6	2
Total moderate	4	3	4	2	4	0	4
Total high	0	0	0	0	1	0	0
Total NA	0	0	0	0	0	0	0

PALS, global longitudinal atrial strain/peak atrial longitudinal strain.

**Table 3:** Risk of bias assessment (RoB) QUIPS outcome 2: pLAct rate (1/s)

Study	Study participation	Study attrition	Prognostic factor measurement	Outcome measurement	Study confounding	Statistical analysis reporting	Overall risk of bias
Hirose et al.2011	moderate risk of bias	low risk of bias	moderate risk of bias	low risk of bias	high risk of bias	low risk of bias	moderate risk of bias
Hong et al. 2013	moderate risk of bias	moderate risk of bias	moderate risk of bias	moderate risk of bias	moderate risk of bias	low risk of bias	moderate risk of bias
Imanishi et al.2013	low risk of bias	moderate risk of bias	low risk of bias	low risk of bias	low risk of bias	low risk of bias	low risk of bias
Total low	2	2	1	3	1	4	1
Total moderate	2	3	3	1	2	0	2
Total high	0	0	0	0	1	0	0
Total NA	0	0	0	0	0	0	0

pLAct, left atrial contraction strain rate.

### Primary outcomes

#### GLAS (%)/PALS

**Figure 2:**
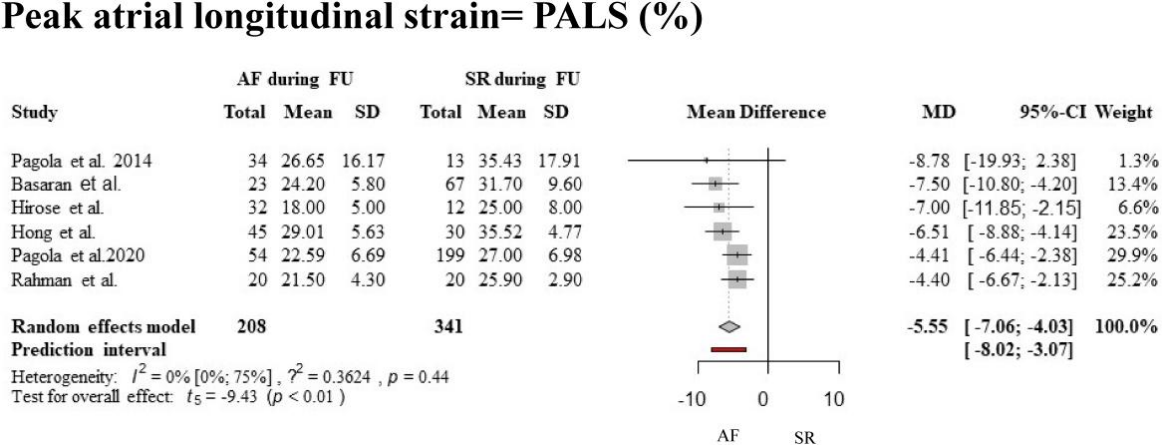
Forest plot with pooled mean and SD values, representing the difference between the baseline PALS of patients with and without future atrial fibrillation. AF, atrial fibrillation; SD, standard deviation.

A total of six^26,28–30,32,33^ studies were eligible for quantitative synthesis regarding the outcome PALS. Based on our results, patients with AF detected during follow-up had significantly reduced baseline PALS values in sinus rhythm, compared with patients who did not develop AF during follow-up as expressed in mean differences of baseline PALS values [WMD (weighted mean difference), −5.55, 95% CI, −7.06 to −4.03, prediction interval, −8.02 to −3.07], see *Figure 2* and *Table 4*.

**Table 4:** Key echocardiographic parameters in the included studies

Study	AF group			Control group					
	LAVi Mean ± SD (mL/m2)	PALS Mean ± SD (%)	pLAct Mean ± SD (1/s)	LAVi Mean ± SD (mL/m2)	PALS Mean ± SD (%)	pLAct Mean ± SD (1/s)	*P*-value LAVI	*P*-value PALS	*P*-value pLAct
Hirose et al. 2011	59 ± 12	18 ± 5	−0.9 ± 0.2	46 ± 16	25 ± 8	−1.4 ± 0.5	<0.001	<0.001	<0.001
Hong et al. 2013	30.12 ± 6.77	29.01 ± 5.63	2.08 ± 0.48	27.53 ± 5.37	35.52 ± 4.77	1.64 ± 0.51	0.082	<0.05	<0.05
Pagola et al. 2014	N/A	26.6 ± 16.17	N/A	N/A	35.43 ± 17.91	N/A	N/A	<0.001	N/A
Pagola et al. 2021	33.5	22.59 ± 6.69	N/A	29	27 ± 6.98	N/A	0.002	0.0001	N/A
Basaran et al. 2015	34 ± 11.3	24.2 ± 5.8	N/A	26.4 ± 8.4	31.7 ± 9.6	N/A	<0.001	<0.001	N/A
Rahman et al. 2014	20.1 ± 5.2	21.5 ± 4.3	N/A	18.3 ± 1.6	25.9 ± 2.9	N/A	<0.0001	<0.001	N/A
Imanishi et al.2013	66 ± 20	N/A	0.78 ± 0.26	58 ± 17	N/A	1.1 ± 0.22	ns.	N/A	0.0029

LAVi, left atrial volume index (mL/m^2^); PALS, peak atrial longitudinal strain (%); pLAct, left atrial contraction strain rate (1/s).

The paper from Lin et al.^31^ also stated that PALS is considerably decreased in those with ongoing atrial fibrillation.

#### pLAct rate – atrial contraction strain rate (1/s)

**Figure 3:**
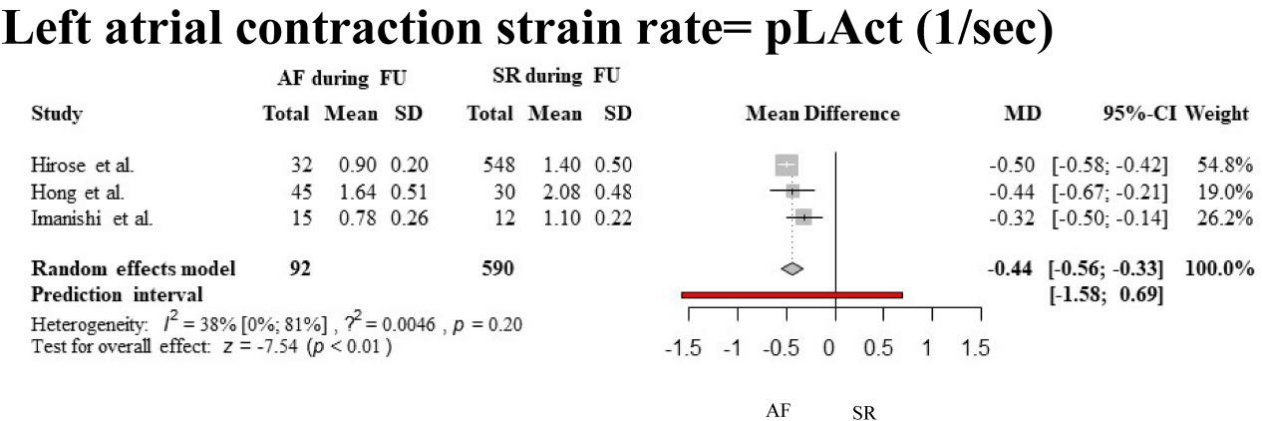
Forest plot with pooled mean and SD values, representing the difference between the baseline pLAct of patients with and without future atrial fibrillation. AF, atrial fibrillation; SD, standard deviation.

Our results based on the quantitative synthesis of three studies^26–28^ showed that pLAct rate is significantly decreased in patients with future AF (WMD −0.44, 95% CI, −0.56 to −0.33, prediction interval −1.58 to −0.69). Our analysis resulted in low heterogeneity (38%, 95% CI 0–81%), see *Figure 3* and *Table 4*. Qualitative assessment of the study of Lin et al. also concluded, that pLAct rate is significantly decreased in those with AF.

### Secondary outcomes

#### pLAc − PACS (%)

Qualitative synthesis of the only two studies^27,30^ reporting pLAct as an outcome showed clearly that the baseline atrial contraction strain parameters differ significantly between the patients with and without future AF.

#### Srs (1/s) and sre (1/s)

According to the results of all the four studies,^26–28,31^ decreased baseline Srs and Sre are associated with the occurrence of AF during follow-up.

#### LA stiffness index (ratio of *E/e*′ to sr)

Both studies^31,33^ reported increased baseline stiffness of the LA wall affecting patients with AF. The findings of Rahman et al. are more relevant for our work, because measurements were performed during sinus rhythm.

## Discussion

### Main findings

This first systematic review and meta-analysis in the topic with eight studies comprising data of 1230 patients revealed a significantly lower value in baseline left atrial strain parameters in patients who developed AF compared to those who remained in sinus rhythm during follow-up. Our findings highly suggest that two-dimensional speckle-tracking echocardiography could be a promising method for the assessment of left atrial strain in a risk population for AF to evaluate the risk of AF.

According to the task force recommendation to standardize deformation imaging,^25^ the most exact value for LA function assessment is PALS, which is a tangential strain measured from the apical four-chamber view, pointing towards the endocardial atrial border. We could also calculate a reliably narrow prediction interval for PALS in our meta-analysis (see *Figure 2*). Considering that LA contraction strain is only present in sinus rhythm, we also aimed to analyse whether it is also decreased in patients who later developed AF during follow-up. Most of the studies reported only contraction strain rate (pLAct rate), which is the time derivative of contraction strain. Hirose et al.^26^ also reported booster pump function with contraction strain rate values in their prospectively designed study, and found that the pLAct rate was the most sensitive predictor of new-onset AF.

### Main observations of the included studies

All included studies emphasized that the decrease in LA strain parameters is independent from changes in left atrial volume index (LAVi).

Two cohort studies^27,30^ included strain measurement in patients before they underwent cardiac surgery [coronary artery bypass grafting (CABG) or aortic valve replacement (AVR)]. Both concluded that the preoperative assessment of LA function could predict the occurrence of postoperative AF (POAF). A multivariate logistic regression analysis performed by Basaran et al.^30^ identified increased PALS as an independent negative predictor of POAF after CABG (OR: 0.839, *P* = 0.013). Imanishi et al.^27^ found decreased pLAct rate as the only independent predictor of POAF after AVR on multivariate analysis (*P* = 0.0221). According to their receiver–operator characteristic (ROC) curve, pLAct rate with a cut-off value of <0.79 1/s predicted AF with a sensitivity of 60%, specificity of 92%, and area under curve (AUC) of 0.828. Noteworthy, 56% of their consecutive patients developed POAF. Implementing these diagnostic tools preoperatively has a promising potential to identify patients at higher risk for POAF and to introduce preventive therapy in time to potentially avoid complications related to the arrhythmia.

Meanwhile Lin et al.^31^ found that AF plays mainly an additive role in decreased atrial performance, which resulted from aging, Pagola et al.^29^ stated that age is only a good predictor when comparing very young stroke patients to elderly ones regarding atrial cardiomyopathy and AF development. In the studies of Hirose et al.^26^ and Basaran et al.,^30^ age heterogeneity was present between the two groups, which may distort the results.

Two studies of Pagola et al.^29,32^ investigated patients with IS. The first study from 2014^32^ highlighted that measuring left atrial volume is not reliable enough in the elderly, because initially significant differences between AF and non-AF patients disappear by aging,^34^ but LA strain assessment is more sensitive and age-independent. The other study by Pagola et al. published in 2020^29^ found that only a long daily paroxysmal AF burden (>5 h) at follow-up came with significantly decreased baseline PALS values, whereas patients with shorter than 5 h of AF duration on a 24 h Holter had comparable mean PALS values with patients with no AF at all (20 vs. 27 vs. 29%, respectively). A PALS cut-off value of 25.3% predicted AF with 70% sensitivity and 60% specificity in this study. When this value was combined with an NT-proBNP cut-off of 283 ng/mL, the prediction model improved to a sensitivity of 79% and specificity of 63%, with an AUC of 0.82.

Longer AF duration increases the risk of thromboembolism according to the observations of Healey et al.in the ASSERT trial where they pointed out that the risk of stroke increased, when the episodes of AF were longer than 18 h.^5^ This shows clearly that the detection of a clinically significant AF burden requires prolonged and expensive heart rhythm monitoring. With the help of a sensitive, non-invasive assessment of LA function we could probably limit the number of patients who would really need it. Hong et al.^28^ emphasized the importance of functional remodelling assessment, which presents before electrical remodelling in first-diagnosed (previously mentioned as lone) AF patients. This key finding alludes to the fact that AF detected by heart rhythm monitoring can only assess the stage where functional remodelling has already been developed.

Based on experimental and clinical observations, Lin et al.^31^ stated that LA fibrosis leads to decreased LA performance with aging, where the surrogate marker for fibrosis is expressed as lower LA voltages on electroanatomical mapping. Based on their results PALS correlated tightly and linearly, whereas LA stiffness index correlated inversely with mean LA voltages (*P* < 0.001). Decreased PALS and voltage values form the ground for the development of AF by mirroring adverse functional-, anatomical and electrophysiological changes of remodelling in the LA.

The findings of all included studies in this analysis suggest that AF might only be the tip of the iceberg, because changes on the cardiomyocyte level and on functional level in the left atrial remodelling develop before the clinical manifestation of the arrhythmia. These align with the findings of the NOAH-AF NET study,^13^ that anticoagulation introduced based on AHRE is not beneficial. LA remodelling is progressing faster and AF burden is usually higher under some conditions, like HT, DM, hyperlipidaemia and heart failure with preserved ejection fraction (HFpEF).^17,35^ Approximately one half of all HFpEF patients develop AF.^35^ As elevated LV filling pressures promotes LA remodelling, LA strain is a surrogate and reliable marker also in this patient population, as its decreased values are correlated with AF in HFpEF patients.^36^ LA strain is useful when added to LAVi when assessing diastolic dysfunction of the left ventricle.^37^

The ARCADIA trial^38^ found, that patients after cryptogenic stroke and ACM in SR do not benefit from the initiation of OAC. However, in this study, ACM was defined as *P*-wave terminal force greater than 5000 μV × ms in ECG lead V1, NT-proBNP greater than 250 pg/mL, or LA diameter index of 3 cm/m^2^ or greater on echocardiogram. These results can be explained by the fact that LA diameter alone is not a sophisticated echocardiographic parameter to assess ACM and a more sensitive diagnostic approach (e.g. LA strain imaging) is warranted. Although our study was underpowered to determine cut-off values of LA strain for AF prediction, we believe, the PALS cut-off value might be around 29% according to a prospective study from Ewelina et al. They found, that HFpEF patients with PALS below 29.4% are at 33-fold greater hazard of AF (*P* < 0.001) compared with those of non-impaired PALS.

We believe, that an improved, structured risk stratification model based on comorbidities, on circulating biomolecules level (NT-proBNP, BMP10, troponin, angiopoietin-2, fibroblast growth factor-23 etc.),^15^ and on advanced non-invasive imaging for detecting even subclinical ACM needs to be established in a prospective manner. Moreover, according to novel directions in electrophysiologiy if once AF is diagnosed, , AF burden needs to be determinted and incorporated in the decision making pathway^15^ to further guide OAC initiation. One really promising part of this future model could be also LA strain assessment using two-dimensional speckle-tracking echocardiography.

### Strength

To the best of our knowledge, this is the first meta-analysis, which highlights the outstanding performance of LA deformation parameters in identifying patients at high risk for AF in the future. We analysed the data of a considerable number of patients despite the low number of studies included in the meta-analysis.

### Limitations

This systematic review and meta-analysis shows all potential limitations, which can possibly come up with such an analysis. Altogether eight articles were analysed; however, six articles were eligible for quantitative PALS analysis, and only three were eligible regarding pLAct rate. Two articles were only eligibile for qualitative analysis. The spectrum of patient population was quite heterogeneous from first-diagnosed (previously mentioned as lone AF) AF patients to IS patients and patients with severe aortic stenosis awaiting operation. There was no uniform data reporting neither in AF monitoring, nor in two-dimensional speckle-tracking echocardiography parameters. We should add that LA strain measurement with speckle-tracking echocardiography seeks some limitations like interobserver variability, noise, and bad reproducibility when using non-automated strain measurement softwares. However, using the QRS as the reference point for the analysis of PALS is superior, in terms of feasibility, reproducibility, and time, compated to P-wave as a reference point.^39^ Nowadays, fully automated softwares for LA strain measurements are available which are proven to carry very low interobserver variability, are easily reproducible and not noisy.^40^ Different studies used various ECG-monitoring methods and follow-up times for AF detection and also the softwares for the offline speckle-tracking analysis were differing among the studies. Some of the studies used symptom-driven AF screening, which could lead to underdiagnosed arrhythmias. Moreover, there was an age heterogeneity among the included studies, and it is well-known that despite no AF development, electrical and structural remodelling can be seen in the elderly.^41^ This physiological alteration might be able distort the results and cause bias. We also have to mention that because of the novelty of the topic, we had a limited number of eligible studies for the analysis; however, they appear quite old as there is no new prospective study in the topic. As we had no access to patient-level data, we could neither perform propensity-score matching to create balanced cohorts in baseline clinical parameters nor were we able to establish a refined prediction model for AF occurrence based on all included patients.

## Conclusion

Our findings suggest that there are significantly reduced baseline LA strain and strain rate parameters measured in sinus rhythm in patients who develop AF during follow-up. Given the nature of this analysis, our study is underpowered to determine cut-off values of LA strain for the initiation or for the withhold of anticoagulation. However, using LA speckle tracking as a diagnostic tool in a future multifactorial prediction model combined with AF burden and comorbidities, could lead to a better and more sophisticated patient selection for OAC or at least for prolonged heart rhythm monitoring to determine AF burden.

### Implication for practice and future research

**Figure 4:**
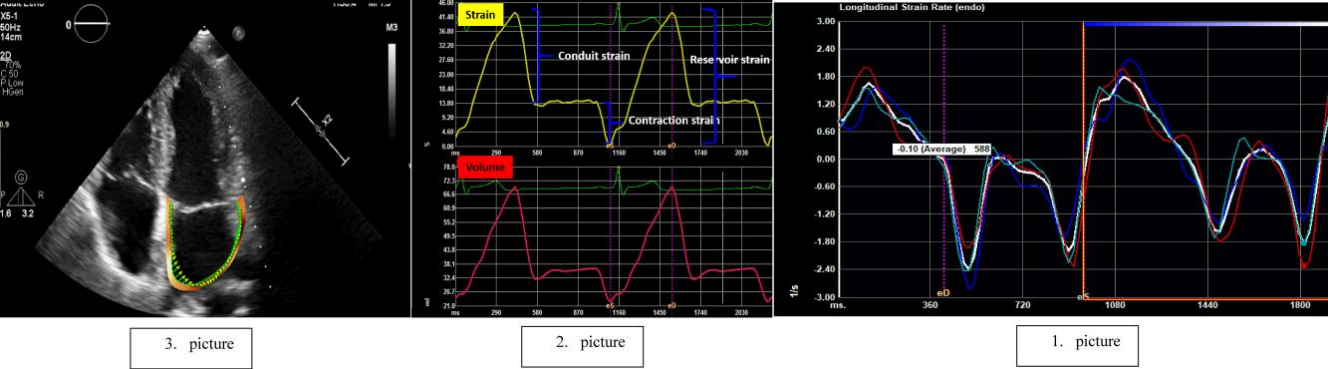
Flowchart of the measurement process (own data from the heart and vascular centre of Semmelweis University). Picture 1 showing a high-quality two-dimensional transthoracic four-chamber view and encountering the LA with TomTec ™ software. Picture 2 showing LA strain measurements (%) with TomTec ™ software. LA strain rate measurement (1/s) with TomTec ™ software.

By implementing such a strategy in everyday practice, we could lower the number of unnecessary AF screenings, which would also lead to better distribution of healthcare costs and resources. To properly use this complementary diagnostic tool in everyday practice, we need to prospectively define LA strain (most probably PALS and pLAct rate) cut-off values (measurement method seen in *Figure 4*) or ranges for patients who need prolonged ECG monitoring. Future RCTs are also warranted to prospectively test the performance of these cut-off values in secondary or even in primary stroke prevention. The results of this meta-analysis can be regarded as a clear call for action in the field.

## Lead author biography


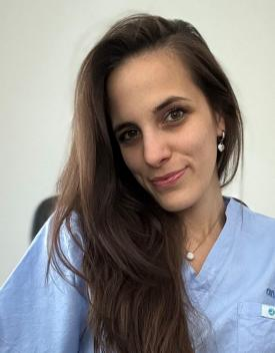


Henriette Mészáros is a cardiologist in training at the Heart and Vascular Centre of Semmelweis University in Budapest, Hungary with a special interest in echocardiography and atrial fibrillation. Her primary focus lies in stroke prevention and the cardiac diagnostic workup following ischaemic stroke, which serves as the basis for this publication. Through her research and clinical practice, she aims to contribute to advancements in cardiovascular imaging and stroke management. This work reflects her commitment to improving diagnostic precision and outcomes for patients with cerebrovascular and concomitant cardiovascular conditions.

## Authors contribution according to ICMJE recommendation

H.M. contributed to conceptualization, project administration, writing the original draft; P.F. contributed to statistical analysis, visualization; R.N. contributed to data curation, methodological guidance, review and edit the original draft; B.P.M., S.N., P.Á. contributed to conceptualization, methodological guidance, supervision, writing the original draft, review, editing; E.P. contributed to selection process, risk of bias assessment; Z.S. contributed to data collection, data table editing; images. P.V. contributed to methodology, risk of bias assessment; A.Á.M. acts as the echocardiographic specialist. All authors have (1) contributed to the concept of the study; (2) revised the manuscript; (3) read and approved the final version of the manuscript.
